# Optimizing growth and yield of striped catfish (*Pangasianodon hypophthalmus)* and quinoa (*Chenopodium quinoa*) in a biosaline integrated aquaculture–agriculture systems

**DOI:** 10.1038/s41598-024-67414-x

**Published:** 2024-07-30

**Authors:** Fahad Kimera, Muziri Mugwanya, Walaa Ahmed, Mahmoud A. O. Dawood, Hani Sewilam

**Affiliations:** 1 Center for Applied Research on the Environment and Sustainability (CARES), School of Science and Engineering, The American University in Cairo, AUC Avenue, P.O. Box 74, New Cairo, 11835 Egypt; 2 Animal Production Department, Faculty of Agriculture, Kafrelsheikh University, Kafr El-Sheikh, Egypt; 3 Department of Engineering Hydrology, Faculty of Civil Engineering, RWTH Aachen University, 52074 Aachen, Germany

**Keywords:** Biosaline, Brackish water, Integrated aquaculture–agriculture system, Quinoa, Striped catfish, Sustainability, Ecology, Plant sciences, Ecology, Environmental sciences

## Abstract

Soil salinity and freshwater scarcity are among the major global threats to sustainable development owing to their adverse impacts on agricultural productivity especially in arid and semi-arid regions. There is a need to find sustainable alternatives such as salt-tolerant crops and fish to improve people’s livelihoods in marginal areas. This study aimed to maximize the growth and yield of striped catfish (*Pangasianodon hypophthalmus*) and quinoa (*Chenopodium quinoa*) cultivated under a biosaline integrated aquaculture–agriculture system. The study was laid in a randomized completely block design of three saline effluent treatments under three replicates: 5000 ppm (T1), 10,000 ppm (T2), 15,000 ppm (T3), and control (T0). Agro-morphological and physiological attributes of quinoa were measured. The crop yield in biomass and mineral element composition was also studied. Additionally, fish growth performance parameters such as feed intake and efficiency, growth, and survival rate were also calculated. Our results indicated that irrigating quinoa with saline aquaculture effluents above 10,000 ppm enhanced the plant growth, yield, and nutrient content of seeds. Furthermore, rearing striped catfish in saline water reaching up to 15,000 ppm did not have adverse impacts on the growth and survival of fish. Overall, integrating catfish and quinoa production under a salinity regime of 10,000 ppm could be a potential solution to ensuring alternative food sources in marginal areas.

## Introduction

The influence of climate change on salt accumulation in soils has become a major concern around the global^1^.This is because salt accumulation in soils causes osmotic stress and ion toxicity in plants leading to a reduction in nutrient absorption and photosynthesis as well as disruption of metabolic activities of plant enzymes^2^. Furthermore, increased soil salinization has been reported to negatively impact the grain yield of crops with percentage losses reaching up to 47%^3^. In the same regard, soil salinization has been reported to reduce photorespiration, decrease protein synthesis, denature cell membranes, and cause the closure of stomata in salt-sensitive plants^4^.The most dangerous effect of salt stress in plants is the production and accumulation of reactive oxygen species (ROS) which lead to ion toxicity by damaging essential components (proteins, lipids, and DNA) in plants^4^.On the other hand, salinity has significant impacts on the structure and physicochemical properties of the soil^5^. For instance, soil salinity has been reported to change the soil structure of clay particles (i.e. clay particles scuttle and their pores close) thus leading to an increase in soil density which causes a reduction in water drainage^6^. As such, accumulation of ions takes place leading to increased soil alkalinity^6^. Changes in the soil physicochemical properties have been shown to influence the abundance and diversity of the soil microbiome^7^, which negatively affects nutrient recycling in salt-affected soils. However, certain soil-dwelling microorganisms can still survive in saline conditions by undergoing salt-tolerant mechanisms hence leading to the accumulation of both organic and inorganic matter which affects the chemical structure of the soil^7,8^. Besides salinity, freshwater scarcity is another global challenge threatening food security in different regions of the world. With the ever-increasing human population (i.e., expected to reach 9.8 billion by 2050)^1^, there is a need to find sustainable alternatives aimed at increasing food production with limited resources. The utilization of underground brackish water for irrigation of salt-tolerant crops and aquaculture production is a promising strategy in safeguarding food security in marginal regions^3^. Therefore, it is imperative to search for crops with high tolerance to salinity for sustainable food production in regions affected by freshwater scarcity.

*Chenopodium quinoa* belongs to the *Chenopodiaceae* family*.* Quinoa is a halophyte plant that grows and completes its cycle under severe conditions by adapting physiologically and morphologically to salinity stress. Quinoa can withstand highly saline conditions reaching up to 45,000 ppm water salinity^9^, and thus a future crop for food security in marginal regions^9^. Moreover, quinoa can absorb salts and heavy metals from the soil and accumulate them in its roots or shoots thus a suitable candidate for crops that could be used in the reclamation of salt-affected and heavy metal-contaminated lands. This can pave the way for the cultivation of salt-sensitive and heavy metal-sensitive crops which in turn will lead to increased food production that meets the current food demand^1,10^. Quinoa is termed as a complete food^11^ due to its incredible nutritional and health benefits, which meet the Food and Agriculture Organization (FAO) standards of human nutritional needs^9,11^. For instance, quinoa leaves and seeds are edible, and these are rich in all nine essential amino acids (phenylalanine, isoleucine, tryptophan, histidine, leucine, lysine, threonine, and valine). As mentioned, quinoa contains lysine, which is rare within the plant kingdom and is essential for human nutrition. Quinoa also provides minerals (calcium, magnesium, potassium, phosphorus, sulfur, sodium, iron, cobalt, zinc, copper, and manganese)^9,11^, vitamins (C, A, B1, B2, B3, B5, B7, and folic acid), soluble sugars, and bioactive compounds (flavonoids, carotenoids, and phenolic compounds) which are antioxidants that have health benefits. Furthermore, quinoa contains a balanced amount of oil, fats (unsaturated fatty acids), proteins, and carbohydrates which are important in human health^1,11^. Therefore, quinoa is a superfood recommended for undernourished and obese people and has been reported to mitigate malnourishment and morbidity in children^1^. Likewise, it was chosen as an ideal crop for the crew in the space missions’ nutritional requirements^12^.

Besides the cultivation of salt-tolerant crops, adaptation, and rearing of freshwater fish species in saline water could also lead to increased food production in marginal regions. Moreover, the integration of aquaculture and agriculture would maximize profits and protect the environment from pollution because of poor disposal of aquaculture nutrient-rich wastewater^13^. Striped catfish (*Pangasianodon hypophthalmus*) is an ideal candidate for inland saline aquaculture due to its economic importance, nutritional value, distribution, high productivity rate, adaptation to severe conditions as it can live and grow in polluted or low-quality water, and disease impedance^14,15^. To the best of our knowledge, no study has so far been conducted on the rearing of striped catfish and the cultivation of quinoa under a biosaline-integrated aquaculture–agriculture system. The aim of our study, therefore, was to elucidate the growth response and yield of striped catfish and quinoa as affected by salinity in an IAAS.

## Materials and methods

### Experimental setup

**Figure 1:**
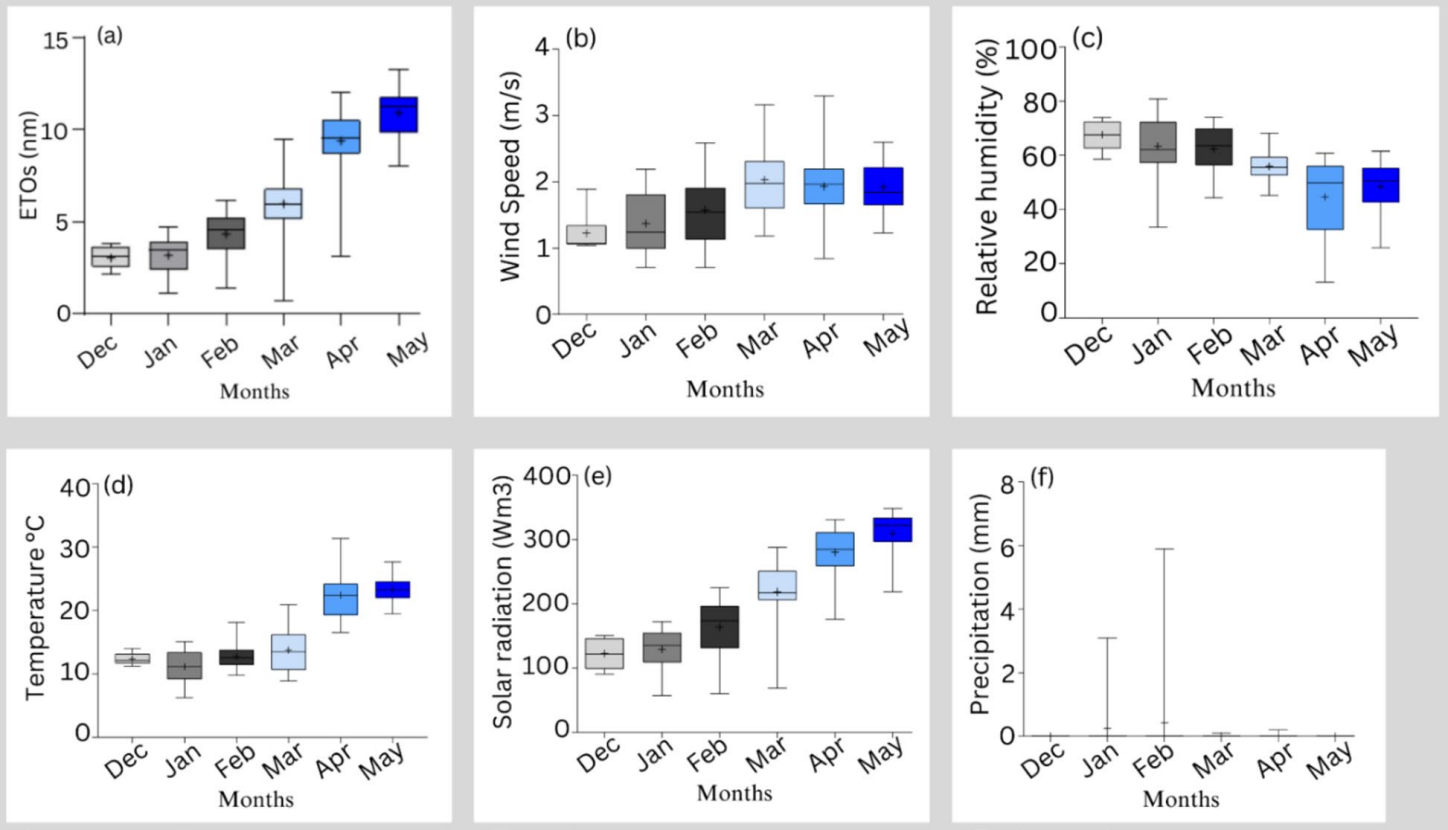
Average weather parameters recorded during the study period. (**a**) Average evapotranspiration, (**b**) average wind speed, (**c**) average relative humidity, (**d**) average temperature, (**e**) average solar radiation, and (**f**) average precipitation. Positive (+) signs indicate the average values of a weather parameter.

A field experiment was conducted from December 2021 to May 2022 at the Center for Applied Research on the Environmental and Sustainability (CARES), The American University in Cairo, New Cairo Egypt (30° 01′ 11.7′′ N31° 29′ 59.8 E) under the following recorded field conditions as shown in Fig. 1. Seeds of quinoa (genotype ICBA-Q3) were obtained from the Agricultural Research Center (ARC) in Giza, Egypt for experimentation.

The experiment was laid out in a randomized completely-block design of three aquaculture wastewater salinity levels under three replications. Striped catfish were cultured in different levels of brackish water salinities and the effluent water was used to irrigate the quinoa crops in the experimental plots. The saline water in the aquaculture tanks was artificially induced by mixing sea salt in freshwater to reach the desired salinity concentrations where the fish were cultivated. The irrigation treatments were as follows: T1—5,000 ppm fish effluents, T2—10,000 ppm fish effluents, T3—15,000 ppm fish effluents, and T0—control (freshwater mixed with inorganic fertilizers). Irrigation was performed using an automated inline dripping system where water was pumped from aquaculture tanks to the experimental plots in an open loop system where the excess water if any, drained in the lower soil layers of the plots. The irrigated water from the tanks was compensated into the tanks twice a week with respective salt concentrations. The chemical properties of the salt used to prepare the different salinity treatments are shown in Table 1. The experimental soil used in this study was analyzed according to standard chemical and physical attributes and presented in Table 2. The soil samples were analyzed at the Agricultural Research Center, Giza, Egypt. The soil particle size distribution was carried out using the pipette method. Electrical conductivity values were measured from the soil paste extract; soil pH values were taken from soil suspensions at a ratio of 1:2.5 as described by Estefan 2013^16^. The available nitrogen in the soil sample was extracted using potassium chloride (KCl) as an extractable solution with the ratio of (5 g soil to 50 ml KCl) and determined using the micro-Kjeldahl method. Available potassium was determined using a flame photometer, and the other elements in the soil sample were determined by using inductively coupled plasma spectrometry (model Ultima 2 JY Plasma) (EPA^17^; Soltanpour^18^, Sewilam et al.^19^).

**Table 1:** Chemical properties of the dry salt sample used to prepare different salinity treatments.

Element	Concentration (%)
NaCl	98.50
SO_4_^2−^	0.31
HCO_3−_	4 × 10^–3^
AsO_4_^3−^	2 × 10^–5^
KI	5.3 × 10^–3^
Mg	0.07
Ca	0.07
Cu	2 × 10^–6^
Fe	3 × 10^–6^
Hg	5 × 10^–6^
Pb	2 × 10^–5^
Cd	8 × 10^–7^
K	0.02
Soluble matter	0.57
Insoluble matter	0.02
Moisture content	0.23

NaCl, sodium chloride; SO_4_^2−^, sulphate; HCO_3_^−^, bicarbonate ion; AsO_4_^3−^, arsenate; KI, potassium iodide; Mg, magnesium; Ca, calcium; Cu, copper; Fe, iron; Hg, mercury; Pb, lead; Cd, cadmium; K, potassium.

**Table 2:** Physical and chemical properties of the soil.

			Anions				Cations			
PH	EC (ppm)	SP	CO_3_^_^ (meq L^−1^)	HCO_3_^_^ (meq L^−1^)	Cl^−^ (meq L^−1^)	SO_4_^_^ (meq L^−1^)	Ca^++^ (meq L^−1^)	Mg^++^ (meq L^−1^)	Na^+^ (meq L^−1^)	K^+^ (meq L^−1^)
7.6	1376	23.00	–	2.36	8.47	11.61	10.61	6.28	5.13	0.43

Available macro and micronutrients (mg kg^−1^)										
		N	P	K	Mn	Zn	Fe	Cu		
		47.00	14.54	48.00	0.43	0.16	1.44	0.06		

EC, electrical conductivity; SP, saturation percentage; CO_3−_, carbonate; HCO_3−_, bicarbonate ion; Cl^−^, chloride; SO_4_^−^, sulfate; Ca^++^, calcium ions; Mg^++^, magnesium ions; Na^+^, sodium ions; K^+^, potassium ions; N, nitrogen; P, phosphorus; K, potassium; Mn, manganese; Zn, zinc; Fe, iron; Cu, copper.

### Growth parameters

Seeds were sown in rows with 30 cm and 50 cm inter and intra-row spacing, respectively. After 2 weeks, weeding was done and the salinity treatments were induced by irrigating plants with different saline aquaculture wastewater treatments (5000 ppm, 10,000 ppm, and 15,000 ppm) and control using a drip irrigation system. The chemical fertilization program (control), plant pests, and diseases were managed according to the recommendations of the Egyptian Ministry of Agriculture.

### Agro-morphological attributes

A total of six plants were randomly tagged within the border per replicate for data collection. Plant heights were measured from the crown to the terminal growing point of the plant using a standard meter rule. Data was collected at four different time points i.e. 30, 60, and 90 days after sowing (DAS). Leaf number and internode number per plant were determined by counting healthy leaves and internodes and calculating averages. Leaf area was calculated according to the equation of^20^ which is given below.

$$Leaf\;area\; = \;L \times W \times C$$

where L is the leaf length, W is the leaf width, and C is the constant (0.74).

Chlorophyll concentration was measured in the early morning before midday using an Apogee Instruments, Inc. Utah, USA MC-100 chlorophyll meter, and the results were reported as SPAD averages. Fresh weights for shoots, roots, and seeds were obtained by weighing the samples in triplicates on a weighing balance, and averages were determined. The samples were then oven-dried to a constant weight at 60 °C for 72 h and dry weights were obtained.

### Seed nutrient composition

Quinoa seed samples were digested in an acid solution with a Berghof microwave digestion system (speed wave Entry DAP-60 K). Briefly, a seed sample of 300 mg was placed into the digestion vessel and 3 ml of 65% nitric acid (HNO_3_) and 35% hydrogen peroxide (H_2_O_2_) were added. The mixture was then carefully shaken and stirred with a clean glass bar for 10 min. The vessel was closed, and the sample was heated in the microwave. After cooling, the resulting clear solution was then used for nutrient composition analysis using an Agilent 4210 MP-AES fitted with a double-pass cyclonic spray chamber and a OneNeb Series 2 nebulizer. Nitrogen was supplied using an Agilent 4107 Nitrogen Generator. All wavelengths were selected from the MP Expert software library according to the sensitivity that was required.

### Fish growth performance parameters

Striped Catfish (initial weight ~ 139.0 g) were stocked at a density of 81 fish per tank. Each tank had a water volume of 1000 L i.e. (11.3 kg fish/m^3^). Fish were cultured for five months and fed on commercial pellets supplied by Skretting Company in the 10th of Ramadan Street, El Sharqia, Egypt. The pellets contained 28% crude protein, 5% crude lipid, 6% crude fiber, 13% ash, and 9% moisture. The feeding pattern and frequency were determined by the fish biomass percentage which ranged from 2 to 3% depending on growth and satiation. The below formula was used to compute fish growth performance characteristics such as feed intake (FI), body weight gain (BWG), feed efficiency ratio (FER), protein efficiency ratio (PER), specific growth rate (SGR), and survival rate.

$${\text{BWG}}\left( {\text{g}} \right) = {\text{Final}}\;{\text{body}}\;{\text{weight}}{-}{\text{initial}}\;{\text{body}}\;{\text{weight}}$$

$${\text{FER}} = {\text{BWG}}/{\text{FI}}$$

$${\text{PER}} = {\text{BWG}}/{\text{protein}}\;{\text{intake}}$$

$${\text{SGR}}\left( \% \right) = \left( {{\text{ln}}\left( {{\text{final}}\;{\text{body}}\;{\text{weight}}} \right){-}{\text{ln}}\left( {{\text{Initial}}\;{\text{body}}\;{\text{weight}}} \right)} \right)/{\text{Experimental}}\;{\text{duration}})$$

$${\text{Survival}}\;{\text{rate}}\left( \% \right) = \left( {{\text{The}}\;{\text{final}}\;{\text{number}}\;{\text{of}}\;{\text{fish}}/{\text{The}}\;{\text{initial}}\;{\text{number}}\;{\text{of}}\;{\text{fish}}} \right)*{1}00$$

### Aquaculture wastewater quality measurements

Water quality parameters such as temperature, pH, and dissolved oxygen were closely monitored using automated digital Nilebot technologies by Conative Labs to fit the ideal required levels for striped catfish. Likewise, water quality parameters such as Ammonia, Ammonium, Ammonia–Nitrogen, Nitrite-Nitrogen, and Nitrate-Nitrogen were quantified once every two weeks until the end of the growing season using water analysis chemical kits from Hanna instruments. Briefly, 100 ml of water was collected from the tanks using a beaker before feeding and immediately taken to the lab for analysis of ammonia, nitrite, and nitrate concentrations using a photometer along with the ammonia reagent kit (H193715-01), nitrite reagent kit (H193707-01), and nitrate reagent kit (H193728-01) from HANNA instruments. The specific absorbance of the nitrogenous elements was measured using an aquaculture photometer device (H183303). The device was set to display the concentrations of ammonia–nitrogen, ammonium, nitrate–nitrogen, and nitrite-nitrogen in mg/L. Real-time analysis results were presented in the manuscript as mean values read from the photometer.

### Statistical analysis

Data analysis was performed using JMP and GraphPad Prism software. Analysis of Variance (ANOVA) was conducted to test for the significant differences among treatments and the Tukey–Kramer test was used to detect the difference in means at α = 0.05. Pearson Correlation Coefficient analysis was performed to detect relationships between different variables. The validity of normality has been checked using the Shapiro–wilk test, and the normal distribution has been tested by D’Agostino & Pearson test (omnibus K2).

### Ethical approval

This study followed the guidelines and approval of the Committee of Animal Welfare and Research Ethics, Faculty of Agriculture, Kafrelsheikh University, Egypt.

### Plant material

All plant materials and related procedures in this study were done in accordance with the guidelines of the Institutional Review Board of the American University in Cairo and the Ministry of Agriculture and Land Reclamation in Egypt.

## Results

### Agro morphological and physiological parameters

**Figure 2:**
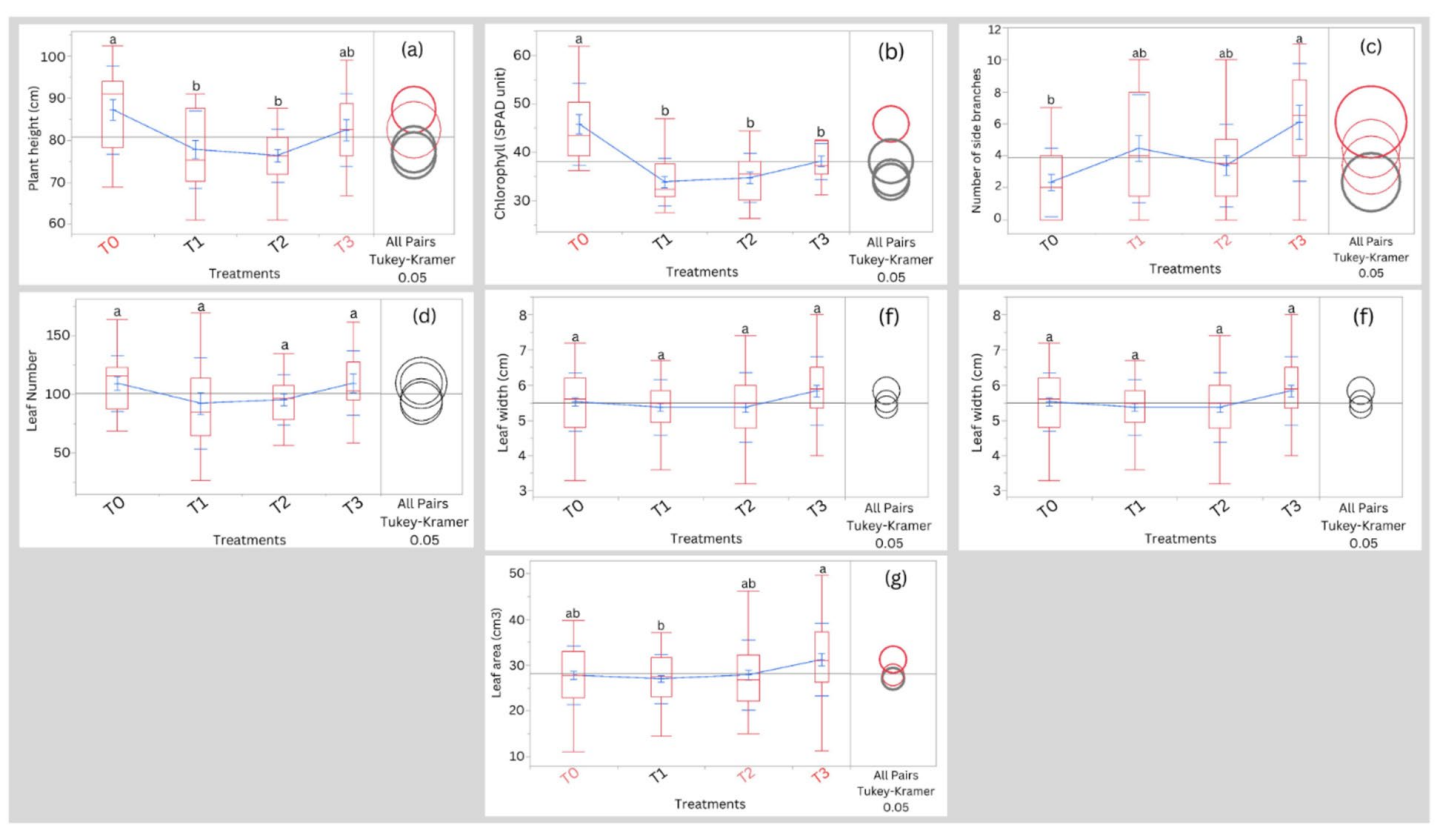
Effect of different treatments on (**a**) plant height, (**b**) chlorophyll content, (**c**) number of side branches, (**d**) leaf number, (**e**) leaf length, (**f**) leaf width, (**g**) leaf area at 75DAS. Treatments having the same letters are not significantly different. Blue lines connect the mean values of different treatments. T0, T1, T2, and T3 represent Control, 5000 ppm, 10,000 ppm, and 15,000 ppm, respectively.

Results on the effect of different treatments on plant height, chlorophyll content, number of side branches, leaf number, leaf length, leaf width, and leaf area at 75 DAS are presented in Fig. 2. At 75 DAS, data on plant height indicated that the treatment T0 significantly (*p* < 0.05) recorded higher values compared to T1 and T2 treatments. However, there were no significant differences in plant heights among the salinity treatments T1, T2, and T3.

**Figure 3:**
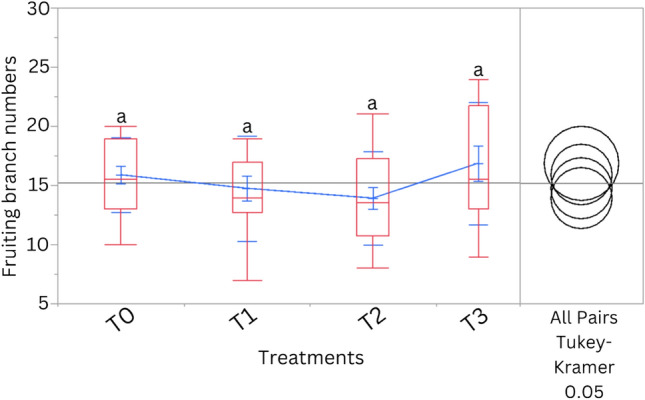
Effect of different treatments on the fruiting branch numbers before harvesting. Treatments having the same letter are not significantly different. Blue lines connect the mean values of different treatments. T0, T1, T2, and T3 represent Control, 5000 ppm, 10,000 ppm, and 15,000 ppm, respectively.

For chlorophyll content, our results showed that the treatment T0 significantly (*p* < 0.05) recorded the highest SPAD values compared to the salinity treatments. Moreover, data on the number of side branches per plant showed significant differences (*p* < 0.05) between treatments T0 and T3, which were later recorded as higher values. For leaf number, leaf length, and leaf width, no significant differences were noted among all the salinity treatments and T0. However, the leaf area in treatment T3 was significantly higher compared to treatment T1. Also, the effect of different salinity treatments and T0 on fruiting branch number before harvesting is shown in Fig. 3.

No significant differences were observed in fruit branching number among the treatments.

**Figure 4:**
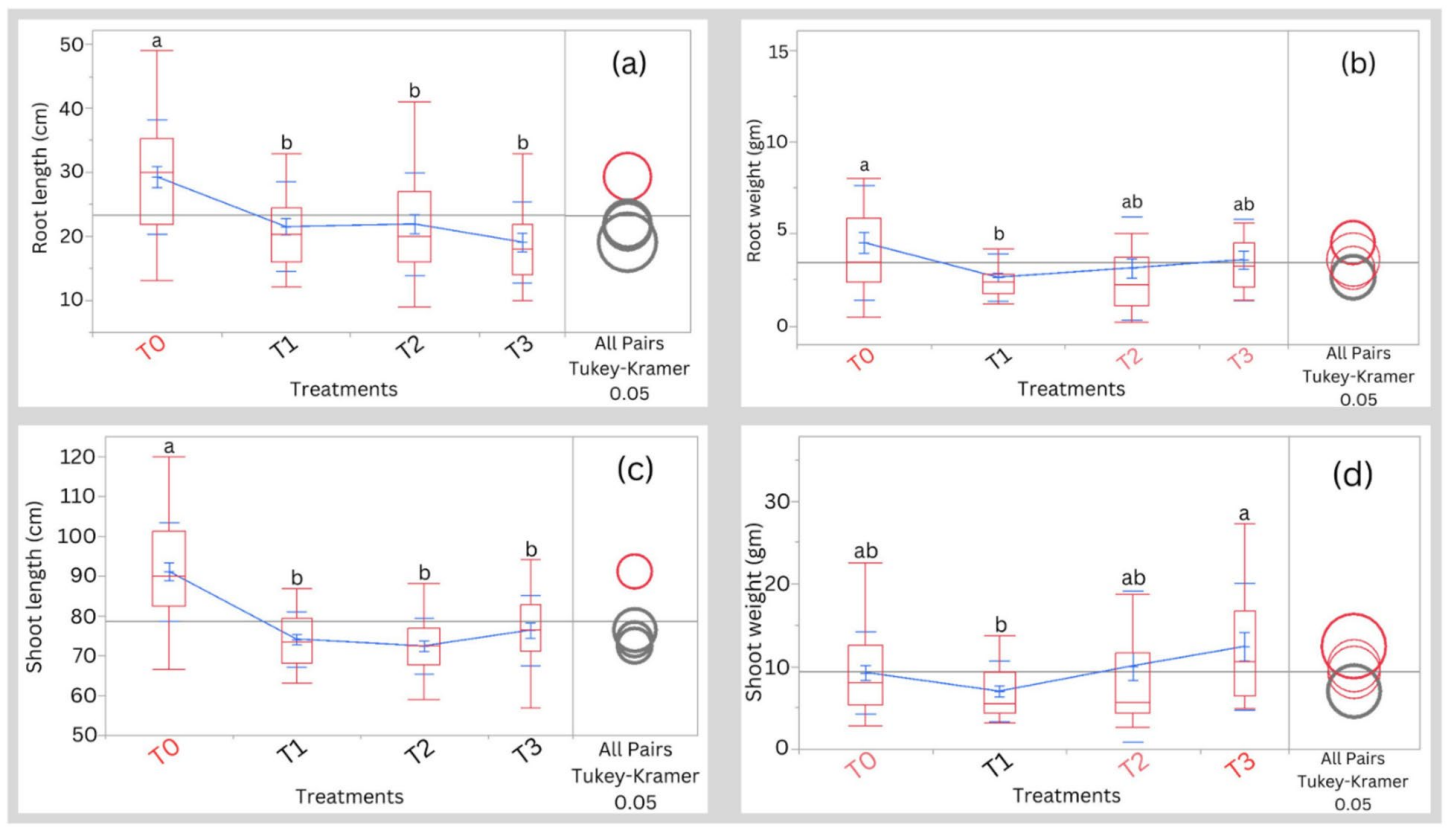
Effect of different treatments on (**a**) root length, (**b**) root weight, (**c**) shoot length, and (**d**) shoot weight at harvesting. Means having the same letter are not significantly different. Blue lines connect the mean values of different treatments. T0, T1, T2, and T3 represent Control, 5000 ppm, 10,000 ppm, and 15,000 ppm, respectively.

Root length, root weight, shoot length, and shoot weight as affected by different salinity treatments and treatments T0 at harvesting are illustrated in Fig. 4. Root length was significantly (*p* ˂ 0.05) higher in the treatment T0 compared to T1, T2, and T3 salinity treatments (Fig. 4a). Data on root weight indicated that treatment T0 significantly (*p* < 0.05) recorded higher values compared to treatment T1 (Fig. 4b). Plants cultivated under the T0 treatment significantly (*p* < 0.05) recorded higher values for shoot length compared to those cultivated under saline conditions (Fig. 4c). Data on shoot weight indicated that the treatment T0 significantly (*p* < 0.05) recorded higher values compared to the T1 salinity treatment (Fig. 4d).

### Crop yield

**Figure 5:**
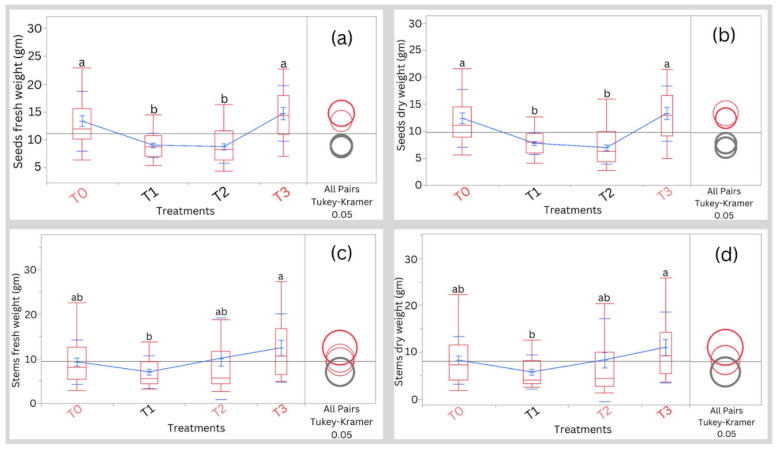
Effect of different treatments on the (**a**) seeds fresh weight, (**b**) seeds dry weight, (**c**) stems fresh weight, and (**d**) stems dry weight at harvesting. Means having the same letter are not significantly different. Blue lines connect the mean values of different treatments. T0, T1, T2, and T3 represent Control, 5000 ppm, 10,000 ppm, and 15,000 ppm, respectively*.*

Figure 5a and b show the seed’s fresh weights and dry weights under different treatments at harvest. At seed fresh weights and dry weights there was a significant difference among the treatments T0, T1, and T2, but no significant difference was noted between the T0 and T3. Also, stem fresh weights and dry weights are presented in Fig. 5c and d. No significant differences in both stem fresh weights and dry weights were noted among treatments T0, T2, and T3. However, the salinity treatment T1 significantly (*p* < 0.05) recorded lower values compared to treatment T3.

### Correlation between agro morphological and Plant yield at harvesting

**Figure 6:**
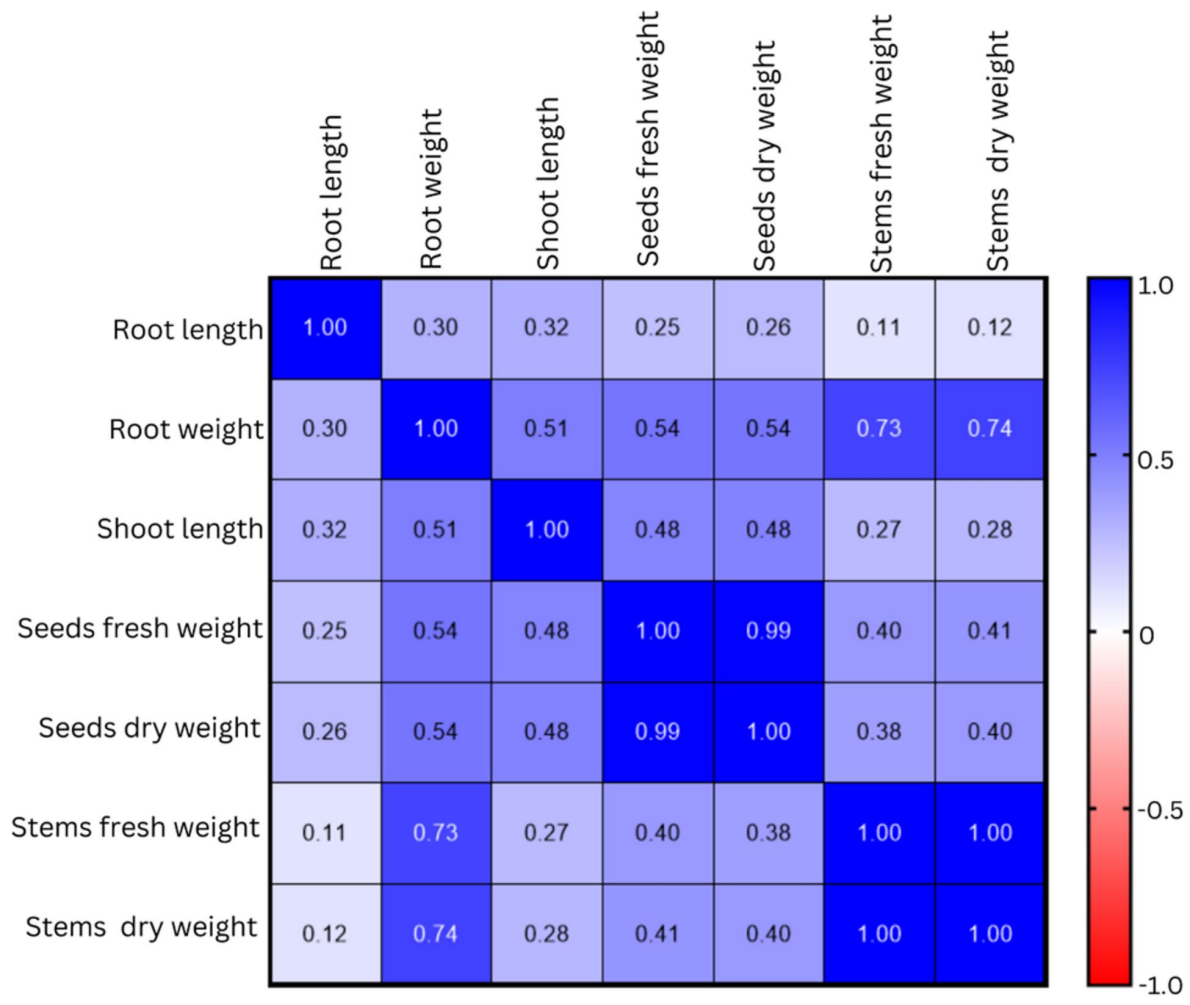
Correlation matrix of agro-morphological and yield parameters of quinoa under different treatments at harvest. Red and blue colors indicate negative and positive significant correlations, respectively, by Pearson Correlation analysis. The color intensity is proportional to the correlation coefficient.

The correlation matrix of agro-morphological parameters of quinoa under different treatments at harvest is presented in Fig. 6. There was no correlation between root length and seeds fresh weights (r = 0.25), seeds dry weights (r = 0.26), stems fresh weights (r = 0.11), and stems dry weights (r = 0.12). Moreover, there was a moderate and positive correlation between root weights, seeds fresh weights (r = 0.54), and seeds dry weights (r = 0.54), but a strong and positive correlation between root weights, stems fresh weights (r = 0.73) and stems dry weights (r = 0.74). There was a weak and positive correlation between shoot length, seeds fresh weights (r = 0.48), and seeds dry weights (r = 0.48). However, there is no correlation between shoot length, stem fresh weights (r = 0.27), and stem dry weights (r = 0.28).

### Nutrient composition of Seeds

Quinoa is rich in different mineral elements including Zinc (Zn), Iron (Fe), Copper (Cu), Manganese (Mn), Calcium (Ca), Magnesium (Mg), Potassium (P), and Table 3 shows the effect of different salinity treatments and control on the nutrient composition of quinoa seeds. Our results indicated no significant differences in nutrient composition among the treatments except for Ca which significantly (*p* < 0.05) decreased in salinity treatments compared to the control (treatment T0).

**Table 3:** Micro and macronutrient elements in quinoa seeds.

Treatments	Zn	Fe	Cu	Mn	Ca	Mg	P
	Mean ± SD	Mean ± SD	Mean ± SD	Mean ± SD	Mean ± SD	Mean ± SD	Mean ± SD
T0	8.81^a^ ± 4.47	107.39^a^ ± 22.08	3.06^a^ ± 1.18	26.66^a^ ± 3.95	1475.94^b^ ± 1794.06	221.47^a^ ± 54.01	0.22 ^a^ ± 0.07
T1	17.51^a^ ± 3.41	99.56^a^ ± 55.31	3.22^a^ ± 1.18	28.64^a^ ± 3.12	359.51^a^ ± 26.65	302.37^a^ ± 21.83	0.24^a^ ± 0.07
T2	9.56^a^ ± 4.56	81.39^a^ ± 11.52	3.03^a^ ± 0.49	22.92^a^ ± 3.12	385.01^a^ ± 11.50	227.41^a^ ± 44.71	0.24 ^a^ ± 0.09
T3	9.16^a^ ± 3.95	82.85^a^ ± 12.01	2.71^a^ ± 0.59	26.86^a^ ± 7.74	407.89^a^ ± 20.38	240.24^a^ ± 33.55	0.55 ^b^ ± 0.01

Zn, zinc; Fe, iron; Cu, copper; Mn, manganese; Ca, calcium; Mg, magnesium; P, phosphorus.

Means having the same superscript letters are not significantly different. T0, T1, T2, and T3 represent Control, 5000 ppm, 10,000 ppm, and 15,000 ppm, respectively.

### Water analysis of aquaculture wastewaters

Aquaculture wastewater quality was determined and the concentration of quality indicators such as ammonia (NH_3_), ammonium (NH_4_), ammonia–nitrogen (NH_3_–N), nitrite (NO_2_^−^), nitrite-nitrogen (NO_2_-N) nitrate (NO_3_^−^), and nitrate-nitrogen (NO_3_-N), across different salinity treatments, were compared and presented in Table 4. Although T2 salinity treatment recorded higher values for NH_3_, NH_4_, and NH_3_–N, no significant differences were noted with other treatments. Likewise, the T3 salinity treatment recorded higher values for NO_2_^−^, NO_2_-N, NO_3_^−^, and NO_3_-N but no significant differences were noted with other treatments. These average values were recorded an hour after feeding the fish but before irrigating crops (before replacing or compensating for new water in the fish tanks).

**Table 4:** Results of the aquaculture wastewater quality at different salinity treatments.

Treatments	Ammonia (mg/L)	Ammonium (mg/L)	Ammonia–nitrogen (mg/L)	Nitrite (mg/L)	Nitrite-nitrogen (mg/L)	Nitrate (mg/L)	Nitrate-nitrogen (mg/L)
	Mean ± SD	Mean ± SD	Mean ± SD	Mean ± SD	Mean ± SD	Mean ± SD	Mean ± SD
T1	2.69 ^a^ ± 2.53	2.84^a^ ± 2.69	2.21^a^ ± 2.09	13.33 ^a^ ± 11.50	4.33^a^ ± 3.51	60.13 ^a^ ± 60.15	13.60^a^ ± 13.60
T2	4.87^a^ ± 4.78	5.16^a^ ± 5.06	4.00^a^ ± 3.93	19.00^a^ ± 16	6.00^a^ ± 5.00	69.70^a^ ± 63.30	15.56^a^ ± 14.50
T3	2.77^a^ ± 2.40	2.92^a^ ± 2.54	2.27^a^ ± 1.98	20.66^a^ ± 14.67	7.33^a^ ± 4.72	87.73^a^ ± 45.25	19.80^a^ ± 10.20

Means having the same superscript letters are not significantly different. T1, T2, and T3 represent 5000 ppm, 10,000 ppm, and 15,000 ppm, respectively.

### Fish growth performance parameters

The effect of different salinity treatments on striped catfish survival rate (SR), body weight gain (BWG), specific growth rate (SGR), and feed efficiency ratio (FER) are presented in Table 5. Although T3 recorded the lowest values for FER, FI, and PER, no significant differences were noted with other treatments (T1, and T2). On the contrary, significant differences were noted in BWG and SGR between treatment T3 as the lowest value and treatment T1 as the highest value. FCR revealed lower values in T1 than in T3 without significant differences with T2. Furthermore, the highest survival rate of fish was recorded in the T2 salinity treatment, however, no significant differences were noted with other salinity treatments.

**Table 5:** Growth performance and survival rate of striped catfish reared under different salinity treatments.

Treatments	FER	BWG	SGR	FI	FCR	PER	Survival Rate
	Mean ± SD (g)						
T1	0.69^a^ ± 0.10	218.17^a^ ± 31.48	0.15^a^ ± 0.01	317.65^a^ ± 2.31	1.46^b^ ± 0.19	2.45^a^ ± 0.26	85%
T2	0.65^a^ ± 0.87	184.87 ^ab^ ± 25.01	0.14 ^ab^ ± 0.01	284.28^ab^ ± 3.75	1.54^ab^ ± 0.28	2.32^ab^ ± 0.31	100%
T3	0.58^a^ ± 0.30	144.70^b^ ± 7.07	0.11^b^ ± 0.005	248.31^b^ ± 4.27	1.72^a^ ± 0.23	2.08^b^ ± 0.27	93%

FER, feed efficiency ratio; BWG, body weight gain; SGR, specific growth rate; FI, feed intake; FCR, feed conversion ratio; PER, protein efficiency ratio.

T1, T2, and T3 represent 5000 ppm, 10,000 ppm, and 15,000 ppm, respectively.

Means having the same superscript letters are not significantly different

## Discussion

Today, the agricultural sector in both arid and semi-arid regions is facing several challenges such as increasing salt accumulation in soils and freshwater scarcity. Here, we study a potential solution for freshwater scarcity. Freshwater scarcity is the major limiting factor in crop and aquaculture production; therefore, we propose the utilization of brackish water as a potential solution in the cultivation and rearing of salt-tolerant crops and fish, respectively. In the current study, irrigating quinoa with water of different salinity concentrations: T0, T1, T2, and T3 did not have a severe effect on the agro-morphological parameters of the plants except for significant differences noted in plant height, chlorophyll content, number of side branches, leaf area, shoot length, root length, root weight, and shoot weight among the treatments (Figs. 2, 3, and 4). We noted that extremely saline condition T3 exhibited similar results to those of the T0 in most parameters except for chlorophyll content, number of side branches, and shoot length. This observation matches the fact that quinoa is a halophyte plant that copes with high salinity conditions but not with lower salinity conditions according to González et al.^9^.

Jaikishun et al.^1^, González et al.^9^, and Hinojosa et al.^4^ reported that quinoa can tolerate salinity concentrations reaching up to 45,000 ppm. In addition, Hinojosa et al.^4^ reported that the optimal concentration for quinoa is approximately 11,000 ppm. However, our study showed that salinity stress decreased the chlorophyll content of quinoa, which is consistent with the findings of Adolf et al.^2^. This is because, under salinity stress, the chloroplasts release reactive oxygen species (ROS) under stress which leads to a decline in chlorophyll concentration in all plants including halophytes^21^. Furthermore, under different salinity treatments, the fresh and dry weights of the seeds and stems were measured (Fig. 5). The seed’s weight dropped dramatically with salinity treatments T1 and T2 for both fresh and dry weights. However, at T3 there was no discernible weight gain above the control.

Toderich et al.^22^, Soltani et al.^23^, and Zeng & Shannon^24^ reported that salinity decreased the seed weight in quinoa, wheat, and rice respectively. However, here we reported that this decline occurred under the lower salinity treatments but not under extremely saline conditions. However, in the stem fresh and dry weights, there were no significant increases in weights in the treatment T0 compared with T2 and T3 salinity treatments, but non-significantly lower values were recorded under treatment T1 compared with T0.

According to previous studies that considered quinoa as a valuable nutrient, we studied the impact of different salinity treatments on the micro and macronutrient concentrations. It was observed in Table 3 that there were no significant differences in most of the mineral elements across all treatments except for calcium. These results matched the findings reported by Koyro & Eisa,^21^. Fe, Cu, and Ca decreased by salt treatment relative to control, it is consistent with the fact that the salt stresses negatively affect their uptake as reported by Nguyen et al.^25^, Karyotis et al.^26^. This explains the reduction in chlorophyll content by salinity because these elements contribute to plant colorimetry^27^, but Mg, P, and Zn increased by salinity because they play a role as salt tolerance enhancers in quinoa^9^. Mn showed nonsignificant changes as it increased under T1 and T3 doses but decreased under T2 like the results reported by Toderich et al.^22^. Calcium response to salinity varies according to species, cell type, or tissue type. The decrease in our case happened as the fact that salinity reduces the ability of plants to take up water and minerals like (Ca) because the osmotic pressure in soil solution surpasses in plant cells under salt stress^25,28^.

The physical, chemical, and biological properties of the water have a major impact on the distribution and activity of aquatic plants and species^28^. Aquaculture animals’ growth, survival, diseases, and feeding rates are all correlated with the water quality^29^. Also, the quality of the water has a major influence on the development and health of plants^30^. There are three forms of nitrogen found in water i.e. ammonia, nitrite, and nitrate^31^. Nitrification bacteria convert ammonia to nitrate because ammonia is one of the most hazardous forms of nitrogen, especially when it’s unionized^29^. Nitrite is not stable since it is a byproduct of the conversion of ammonia or ammonium to nitrate^31^. When compared to the nitrite and ammonia forms, Table 4. Results of the aquaculture wastewater quality at different salinity treatments. Means having the same superscript letters are not significantly different. T1, T2, and T3 represent 5000 ppm, 10,000 ppm, and 15,000 ppm, respectively. Clearly shows that nitrate is the greatest product. Treatment T3 had the highest value, followed by treatment T2, and treatment T1 had the lowest; nonetheless, there is no discernible difference in the nitrate value between the treatments. The fish growth performance parameters (FER, BWG, and SGR) in Table 5 are influenced by the nitrite level, with treatment T1 exhibiting the highest value of fish parameters and treatment T3 displaying the lowest value. It is in line with studies showing that higher nitrate levels in fish bodies change the food intake, rate of growth, and capability to bind oxygen in their blood^32^. Additionally, they cause the fish’s blood’s hemoglobin to change into methemoglobin, which results in the fish’s gills and blood becoming brown and making breathing hard^32^.

The various salinity treatments had no discernible impact on striped catfish growth performance and survival (Table 5) which matched the findings of Hieu et al.^33^ and (Kumar & Chadha (n.d.)^34^. Therefore, the study suggests that striped catfish is tolerant of salt stress. According to Table 5, the survival rate under T2 salinity treatment was 100% but decreased non-significantly under T1 (85.2%) and T3 (93.8%), the findings of Küçük et al.^35^, Djiba et al.^36^, and Zajdband^37^ supported ours, where the survival rate also decreased under the high-salinity treatments. It was also observed a decrease in fish growth performance in terms of the body weight gain (BWG) and specific growth rate (SGR) gradually decreased under extremely saline conditions T3, with significant differences between T1 and T3; however, the feed efficiency ratio (FER) decreased non-significantly under the different salt concentrations. Similar results were reported by Küçük et al.^35^, Abdelrhman et al.^38^, and Wang et al.^8^. Further, an increased feed conversion ratio (FCR) and decreased protein efficiency ratio (PER) were observed in T3. The reason behind BWG, SGR, FER, and PER reduction, and increased FCR under high salinity refers to the lipids, proteins, and carbohydrates catabolism, which increased to generate the energy required for boosting fish tolerance as reported by Toderich et al.^22^ and Kimera et al.^39^.

## Conclusion

To recapitulate, the current study reports that irrigating quinoa with saline aquaculture wastewater at or above 10,000 ppm is efficient for its growth, yield, and nutrient composition. Additionally, the study recommends rearing striped catfish with a maximum water salinity of 10,000 ppm since it showed high survival rates compared to other higher salinity treatments. So, we conclude that, in areas where freshwater is scarce using an integrated system between aquaculture and agriculture by using the wastewater from striped catfish tanks to irrigate quinoa under saline conditions reaching up to 10,000 ppm will be an effective approach to ensuring food security in marginal areas. For further research, the study recommends that this integrated system be applied to all halophytes because we assume that they will respond as same as quinoa which is used as a model for salinity-tolerant crops. Also studying the possibility of applying the IAA system on a broader scale is recommended by using different plants and other salt-tolerant fish in the system.

## Data Availability

The datasets generated during and/or analyzed during the current study are available from the corresponding author upon reasonable request.

## References

[1] Jaikishun, S., Li, W., Yang, Z. & Song, S. Quinoa: In perspective of global challenges. *Agronomy* **9**(4), 176. 10.3390/agronomy9040176 (2019). 10.3390/agronomy9040176

[2] Adolf, V. I., Jacobsen, S. E. & Shabala, S. Salt tolerance mechanisms in quinoa (*Chenopodium quinoa Willd.*). *Environ. Exp. Bot.* **92**, 43–54. 10.1016/j.envexpbot.2012.07.004 (2013). 10.1016/j.envexpbot.2012.07.004

[3] Gleick, P. H. & Cooley, H. Freshwater scarcity. *Annu. Rev. Environ. Resour.*10.1146/annurev-environ-012220 (2021). 10.1146/annurev-environ-012220

[4] Hinojosa, L., González, J. A., Barrios-Masias, F. H., Fuentes, F. & Murphy, K. M. Quinoa abiotic stress responses: A review. *Plants* **7**(4), 106. 10.3390/plants7040106 (2018). 30501077 10.3390/plants7040106 PMC6313892

[5] Choudhary, O. P. *Soil salinity and sodicity land evaluation view project soil quality view project*. https://www.researchgate.net/publication/327824188 (2018)

[6] Yan, N., Marschner, P., Cao, W., Zuo, C. & Qin, W. Influence of salinity and water content on soil microorganisms. *Int. Soil Water Conserv. Res.* **3**(4), 316–323. 10.1016/j.iswcr.2015.11.003 (2015). 10.1016/j.iswcr.2015.11.003

[7] Vilcacundo, R. & Hernández-Ledesma, B. Nutritional and biological value of quinoa (*Chenopodium quinoa Willd.*). *Curr. Opin. Food Sci.* **14**, 1–6. 10.1016/j.cofs.2016.11.007 (2017). 10.1016/j.cofs.2016.11.007

[8] Wang, J.-Q., Lui, H., Po, H. & Fan, L. Influence of salinity on food consumption, growth and energy conversion efficiency of common carp (*Cyprinus carpid*) fingerlings. *Aquaculture* **148**, 115 (1997). 10.1016/S0044-8486(96)01334-8

[9] González, J. A. *et al.* A long journey of CICA-17 quinoa variety to salinity conditions in Egypt: Mineral concentration in the seeds. *Plants* **10**, 407. 10.3390/plants1002 (2021). 33671519 10.3390/plants1002 PMC7926718

[10] Hariadi, Y., Marandon, K., Tian, Y., Jacobsen, S. E. & Shabala, S. Ionic and osmotic relations in quinoa (*Chenopodium quinoa Willd.*) plants grown at various salinity levels. *J. Exp. Bot.* **62**(1), 185–193. 10.1093/jxb/erq257 (2011). 20732880 10.1093/jxb/erq257 PMC2993909

[11] Martínez, E. A., Fuentes, F. F. & Bazile, D. History of quinoa: Its origin, domestication, diversification, and cultivation with particular reference to the Chilean context. In *Quinoa: improvement and sustainable production* 19–24 (Wiley, 2015). 10.1002/9781118628041.ch2.

[12] Ruiz, K. B. *et al.* Quinoa—A model crop for understanding salt-tolerance mechanisms in halophytes. *Plant Biosyst.* **150**(2), 357–371. 10.1080/11263504.2015.1027317 (2016). 10.1080/11263504.2015.1027317

[13] Leogrande, R. & Vitti, C. Use of organic amendments to reclaim saline and sodic soils: A review. *Arid Land Res. Manag.* **33**(1), 1–21. 10.1080/15324982.2018.1498038 (2019). 10.1080/15324982.2018.1498038

[14] Balami, S., & Paudel, K. A review: Use of probiotics in striped catfish larvae culture Molecular metabarcoding of harmful algae as a tool to protect the Scottish aquaculture industry View project Promoting Local Fish Diversity in Selected Lakes of Chitwan through Eco-system-based Comanagement Practices (Supported by UNDP GEF Small Grants Programme of (2022).

[15] Senthilkumaran, B. & Kar, S. Advances in reproductive endocrinology and neuroendocrine research using catfish models. *Cells* **10**(11), 2807. 10.3390/cells10112807 (2021). 34831032 10.3390/cells10112807 PMC8616529

[16] Estefan, G., Sommer, R., & Ryan J. Methods of soil, plant, and water analysis: a manual for the West Asia and North Africa Region: Third Edition, *ICARDA*, (2013)

[17] W. D. O. Thomas A. Office of Research and Development, Methods for the determination of metals in environmental samples, in *EPA* (1991).

[18] Soltanpour, P. N. Determination of nutrient availability and elemental toxicity by AB-DTPA soil test and ICPS. In *Advances in Soil Science* (Springer, 1991).

[19] Sewilam, H., Kimera, F. & Nasr, P. Water energy food nexus model: An integrated aqua-agriculture system to produce tilapia and sweet basil using desalinated water. *Environ. Sci. Pollut. Res.* **30**(6), 15975–15990. 10.1007/s11356-022-23240-0 (2023). 10.1007/s11356-022-23240-0 PMC9908654 36178649

[20] Cheyed, S. H., & Dawood, A. A. *Microgametogenesis tolerant to heat stress in some maize crosses*. https://www.researchgate.net/publication/350789084

[21] Koyro, H. W. & Eisa, S. S. Effect of salinity on composition, viability and germination of seeds of *Chenopodium quinoa Willd*. *Plant Soil* **302**(1–2), 79–90. 10.1007/s11104-007-9457-4 (2008). 10.1007/s11104-007-9457-4

[22] Toderich, K. N. *et al.* Differential impact of salinity stress on seeds minerals, storage proteins, fatty acids, and squalene composition of new quinoa genotype, grown in hyper-arid desert environments. *Front. Plant Sci.*10.3389/fpls.2020.607102 (2020). 33365043 10.3389/fpls.2020.607102 PMC7750330

[23] Soltani, A., Gholipoor, M. & Zeinali, E. Seed reserve utilization and seedling growth of wheat as affected by drought and salinity. *Environ. Exp. Bot.* **55**(1–2), 195–200. 10.1016/j.envexpbot.2004.10.012 (2006). 10.1016/j.envexpbot.2004.10.012

[24] Zeng, L. & Shannon, M. C. Effects of salinity on grain yield and yield components of rice at different seeding densities. *Agron. J.* **92**, 418 (2000). 10.2134/agronj2000.923418x

[25] Nguyen, T. K. H. *et al.* Effects of salinity on growth performance, survival rate, digestive enzyme activities and physiological parameters of striped catfish (*Pangasianodon hypophthalmus*) at larval stage. *Can Tho Univ. J. Sci.* **13**(Aquaculture), 1–9. 10.22144/ctu.jen.2021.011 (2021). 10.22144/ctu.jen.2021.011

[26] Karyotis, T., Iliadis, C., Noulas, C. & Mitsibonas, T. Preliminary research on seed production and nutrient content for certain quinoa varieties in a saline-sodic soil. *J. Agron. Crop Sci.* **189**(6), 402–408. 10.1046/j.0931-2250.2003.00063.x (2003). 10.1046/j.0931-2250.2003.00063.x

[27] Nguyen, P. T. H., Do, H. T. T., Mather, P. B. & Hurwood, D. A. Experimental assessment of the effects of sublethal salinities on growth performance and stress in cultured tra catfish (*Pangasianodon hypophthalmus*). *Fish Physiol. Biochem.* **40**(6), 1839–1848. 10.1007/s10695-014-9972-1 (2014). 25139325 10.1007/s10695-014-9972-1

[28] Zamann, M., Shahidd, S. A. & Heng, L. *Guideline for salinity assessment mitigation and adaptation using nuclear and related techniques* (Springer, 2018).

[29] Kumar Verma, D., Kumar Maurya, N. *Important water quality parameters in aquaculture: An overview Parvind Kumar*. https://www.researchgate.net/publication/362667844

[30] Abd El-Hack, M. E. *et al.* Effect of environmental factors on growth performance of Nile tilapia (*Oreochromis niloticus*). *Int. J. Biometeorol.* **66**(11), 2183–2194. 10.1007/s00484-022-02347-6 (2022). 36044083 10.1007/s00484-022-02347-6 PMC9640449

[31] Omer, N. H. *Water quality parameters*. www.intechopen.com

[32] Putra, I. *et al.* Effect of different biofloc starters on ammonia, nitrate, and nitrite concentrations in the cultured tilapia *Oreochromis niloticus* system. *F1000Res* **9**, 293. 10.12688/f1000research.22977.1 (2020). 32509278 10.12688/f1000research.22977.1 PMC7241270

[33] Hieu, D. Q. *et al.* Salinity affects growth performance, physiology, immune responses and temperature resistance in striped catfish (*Pangasianodon hypophthalmus*) during its early life stages. *Fish Physiol. Biochem.* **47**(6), 1995–2013. 10.1007/s10695-021-01021-9 (2021). 34708321 10.1007/s10695-021-01021-9

[34] Kumar, A., & Chadha, N. K. Salinity tolerance of *Pangasianodon hypophthalmus* in inland saline water: Effect on growth, survival and haematological parameters hormonal manipulation on testicular maturation and spermiation of *Clarias magur* (Hamilton 1822) View project Nutritional requirements & Feeding strategies View project’. https://www.researchgate.net/publication/312777447

[35] Küçük, S., Karul, A., Yildirim, Ş & Gamsiz, K. Effects of salinity on growth and metabolism in blue tilapia (*Oreochromis aureus*). *Afr. J. Biotechnol.* **12**(19), 2715–2721. 10.5897/AJB12.1296 (2013). 10.5897/AJB12.1296

[36] Djiba, P. K. *et al.* Correlation between metabolic rate and salinity tolerance and metabolic response to salinity in grass carp (*Ctenopharyngodon idella*). *Animals* **11**(12), 3445. 10.3390/ani11123445 (2021). 34944222 10.3390/ani11123445 PMC8697877

[37] Zajdband, A. D. *Integrated agri-aquaculture systems* 87–127 (Springer, 2011). 10.1007/978-94-007-1521-9_4.

[38] Abdelrhman, A. M., Sharawy, Z. Z., Goda, A. M. A. S. & Slater, M. J. Adaptability of the Nile tilapia, *oreochromis niloticus* juveniles to water salinity by controlling dietary sodium chloride levels. *Egypt. J. Aquat. Biol. Fish* **24**(2), 225–237. 10.21608/EJABF.2020.86056 (2020). 10.21608/EJABF.2020.86056

[39] Kimera, F., Mugwanya, M., Dawood, M. & Sewilam, H. Growth response of kale (*Brassica oleracea*) and Nile tilapia (*Oreochromis niloticus*) under saline aqua-sandponics-vegeculture system. *Sci. Rep.*10.1038/s41598-023-29509-9 (2023). 36765067 10.1038/s41598-023-29509-9 PMC9913015

